# Low-Oxygen Atmosphere and its Predictors among Agricultural Shallow Wells in Northern Thailand

**DOI:** 10.1016/j.shaw.2014.10.005

**Published:** 2014-10-28

**Authors:** Gobchok Wuthichotwanichgij, Alan F. Geater

**Affiliations:** 1 Office of Disease Prevention and Control 9, Phitsanulok, Thailand; 2 Epidemiology Unit, Faculty of Medicine, Prince of Songkla University, Hatyai, Songkhla, Thailand

**Keywords:** agricultural shallow well, carbon dioxide level, confined space, Northern Thailand, oxygen level

## Abstract

**Background:**

In 2006, three farmers died at the bottom of an agricultural shallow well where the atmosphere contained only 6% oxygen. This study aimed to document the variability of levels of oxygen and selected hazardous gases in the atmosphere of wells, and to identify ambient conditions associated with the low-oxygen situation.

**Methods:**

A cross-sectional survey, conducted in June 2007 and July 2007, measured the levels of oxygen, carbon monoxide, hydrogen sulfide, and explosive gas (percentage of lower explosive limit) at different depths of the atmosphere inside 253 wells in Kamphaengphet and Phitsanulok provinces. Ambient conditions and well use by farmers were recorded. Carbon dioxide was measured in a subset of wells. Variables independently associated with low-oxygen condition (<19.5%) were identified using multivariate logistic regression.

**Results:**

One in five agricultural shallow wells had a low-oxygen status, with oxygen concentration decreasing with increasing depth within the well. The deepest-depth oxygen reading ranged from 0.0% to 20.9%. Low levels of other hazardous gases were detected in a small number of wells. The low-oxygen status was independently associated with the depth of the atmosphere column to the water surface [odds ratio (OR) = 13.5 for 8–11 m vs. <6 m], depth of water (OR = 0.17 for 3–<8 m vs. 0–1 m), well cover (OR = 3.95), time elapsed since the last rainfall (OR = 7.44 for >2 days vs. <1 day), and location of well in sandy soil (OR = 3.72). Among 11 wells tested, carbon dioxide was detected in high concentration (>25,000 ppm) in seven wells with a low oxygen level.

**Conclusion:**

Oxygen concentrations in the wells vary widely even within a small area and decrease with increasing depth.

## 1 Introduction

Persons entering confined spaces are at risk of a variety of health hazards. These hazards stem from the restricted volume of space inside such spaces and the small dimensions of the opening to the outside environment, with consequent difficulty in obtaining access to and exiting from the space, or a combination of the two. These features readily predispose those entering a confined space to exposure to changes in the atmospheric composition within that space, which may be harmful to their health. Confined spaces are most commonly encountered in work situations, and include sewers, casings, tanks, silos, vaults, tunnels, and compartments of ships [1–4].

A common atmospheric change occurring in confined spaces may involve a reduction in the volume percent of oxygen, as a result of oxygen-consuming reactions within the space, replacement of oxygen by other gases, or build-up of toxic gases. Persons entering a confined space may be incapacitated or may even die as a result of these atmospheric changes. While normal atmosphere close to earth contains 20.9% of oxygen by volume, the Occupational Safety and Health Administration regulation and many confined-space guidance documents indicate that an atmospheric oxygen concentration of 19.5% is the lowest level acceptable for entry into such spaces [2,3].

The problems of adverse effects on the health of a person inside a confined space are frequently compounded by the difficulty of making a rapid exit, the difficulty faced by rescuers in gaining entry, and the hindrances to communication between inside and outside of the space.

A number of investigation reports have given data on fatalities in confined spaces. In the United States of America, the Division of Safety Research of the National Institute for Occupational Safety and Health is currently conducting the Fatal Accident Circumstances and Epidemiology project, which focuses primarily on selected electrical- and confined space-related fatalities [5,6]. Suruda et al. [7] analyzed data from the National Traumatic Occupational Fatalities surveillance system, which the National Institute for Occupational Safety and Health had assembled. During the 10-year period between 1980 and 1989, 585 separate fatal incidents occurred in confined spaces, claiming 670 victims. Seventy-two (12%) of the fatal incidents involved multiple victims.

**Fig. 1:**
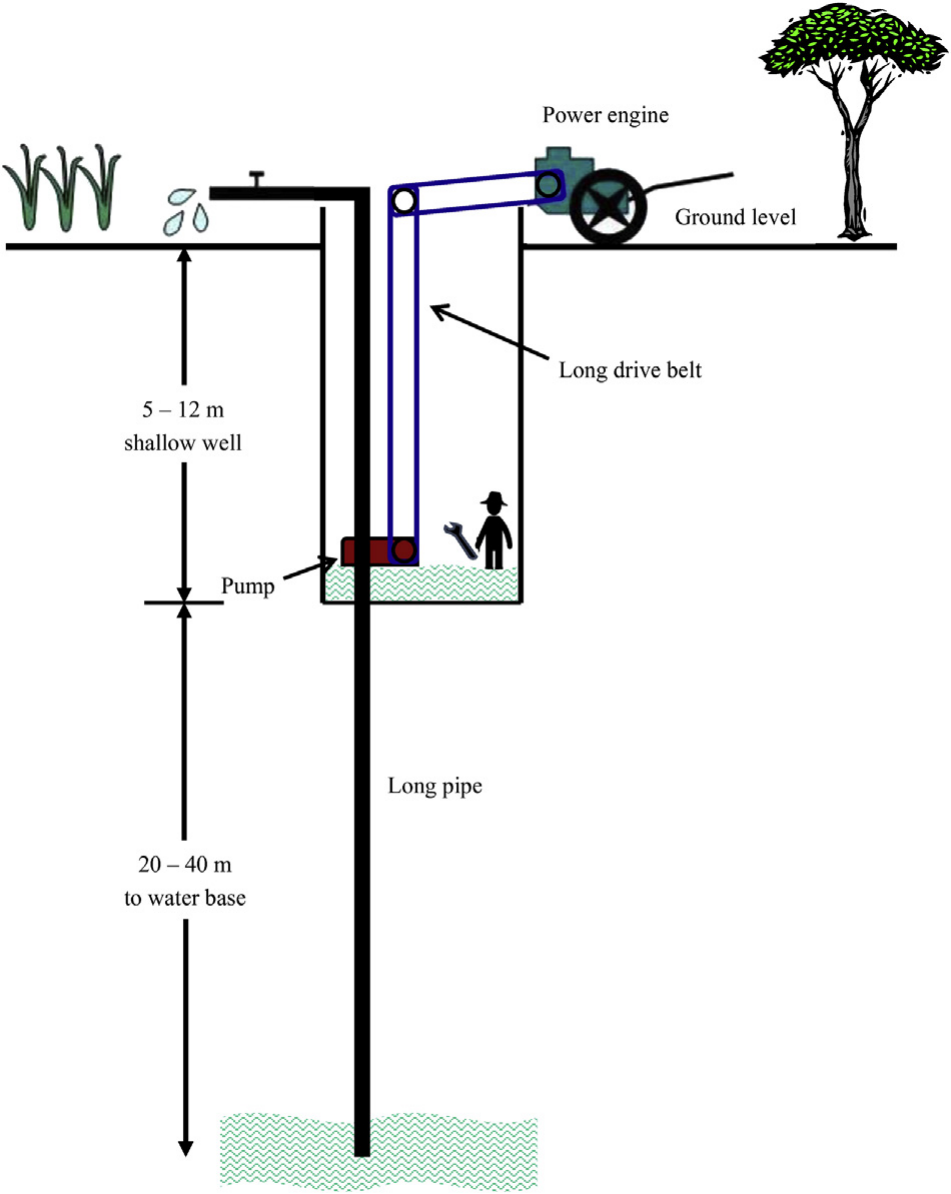
Typical design of an agricultural shallow well in the study region.

In Thailand, cases of death in confined space occur almost every year. Between 2004 and 2008, there were eight investigation reports of 34 deaths and injuries in confined spaces [8]. An estimated 60% of the fatalities involved would-be rescuers. These rescuers had tried to help someone who had collapsed, but presumably had not given due consideration to the reason for the collapse. In one event, in 2006, three farmers died in a shallow well in a field in Kamphaengphet province, northern Thailand. According to the investigation report, local health officers who measured the gas content of the atmosphere at the bottom of the well 6 days later found the oxygen level to be only 5.9% [9]. The Bureau of Occupational and Environmental Diseases, Ministry of Public Health, then measured oxygen levels in 57 other shallow wells in the same region and found that 16% had low levels of oxygen (0.3–16%), which would have posed a hazard to anyone entering them, although toxic gases were not detected in any of the wells [10]. It was reported that the wells were used as a source of irrigation water for paddy fields, and farmers claimed that they had to enter the wells from time to time to set up the pumping system and to carry out general maintenance (Fig. 1).

While irrigation wells are a common feature of the rural landscape in the lower north of Thailand, the survey in Kamphaengphet province [10] made no mention of why some wells presented hazardous conditions while others in the same locality did not. An understanding of the factors predisposing to hazardous conditions in shallow wells would be useful in identifying unsafe wells, and possibly in identifying ways to remediate the condition of hazardous wells. Such understanding may enable the formulation of practical recommendations or guidelines for the safe use and management of shallow wells, for dissemination among farmers and other well users.

This study was therefore conducted with the aim of documenting the variability in oxygen content and occurrence of selected hazardous gases in the atmosphere within shallow wells in the lower north of Thailand, and identifying the ambient conditions—well-construction material and design, frequency of water use, climate, and surroundings—that could predict the low-oxygen condition.

## 2 Materials and methods

A cross-sectional study of shallow agricultural wells in five villages in Kamphaengphet and Phitsanulok provinces, in the lower north of Thailand, was undertaken. Three villages were selected from Kamphaengphet province where the recent fatalities occurred. Two villages were selected from Phitsanulok province based on similarity of agricultural practices to those of the Kamphaengphet villages. Phitsanulok province borders Kamphaengphet province, and the two provinces are similar in that both have many shallow wells that are used for rice-field irrigation.

As distributions of the parameters to be estimated were almost totally unknown, a rigorous estimation of the required sample size was not possible. However, an approximate number was calculated based on the precision of estimating the proportion of agricultural shallow wells with oxygen measurements below a “safe” level (considered here to be 19.5%) [2,3]. Based on the small survey conducted by the health officers from the Bureau of Occupational and Environmental Diseases in Kamphaengphet province, 16% of wells had low-oxygen readings. Estimation of this percentage with a 95% confidence interval (CI) of ±5 percentage points would require a sample size of 207, assuming no missing or unusable data. To allow for 20% wells with unusable data, the planned sample size was raised to 207/0.8 = 259. All wells in four villages were included in the study, and the sample size was augmented by wells from the fifth village. Ultimately, 295 agricultural shallow wells were visited for inclusion as potential study units.

Each of the 295 wells was visited between 08:00 hours and 19:00 hours during June 2007 and July 2007, which falls at the end of the dry season. Forty-two of these wells were almost full of water, with <2 m depth of air column, measured from the top of the surrounding wall, and were excluded from the study sample. Atmospheric composition of the remaining 253 shallow wells was measured using a standardized protocol.

Measurements were made using a portable confined space gas meter (EntryRAE PGM-3000 Confined Space Gas Meter 4-gas; RAE Systems, Inc., San Jose, CA, USA), which can measure the levels of O_2_, CO, H_2_S, and %LEL. This latter measurement represents the concentration of combustible gases as a percentage of lower explosive limit. A 12-m long “Tygon” tubing (internal diameter 0.55 cm) was attached to the inlet of the gas monitor, and the weighted end was lowered into the well atmosphere to take readings at each 1 m depth measured from the top of the surrounding wall of the well. The dead space of the Tygon tubing was calculated to be approximately 285 cm^3^, and the airflow of the monitor was set at 300 cm^3^/minute. At each depth, the monitor was allowed to pump for 1 minute prior to taking a reading, to ensure clearance of the dead space and stabilization of the reading.

A deepest-depth measurement was made at 30 cm above the water surface. Following this, the water was agitated by rapidly dunking a specimen jar into the water 10 times to a depth of around 15 cm prior to collecting a water specimen, to observe and record the color and odor. Then the deepest-depth atmosphere measurement was repeated. Data on ambient conditions (climate, surrounding soil type, other surrounding features, well-construction material and design, presence, type and position of pumping system) were observed and recorded.

Owners of each well were subsequently interviewed to obtain information on the use of the well (purpose, well age, frequency of use, frequency and timing of well maintenance work, type of pumping system, etc.) and the information was recorded on a data form.

The date and time of day of monitoring of each well, as well as the geographic coordinates obtained using a geographic positioning device [Garmin eTrex Vista C; Garmin (Asia) Corporation, New Taipei City, Taiwan] set to show coordinates on the Transverse Mercator System, were recorded. Geographic coordinates were subsequently transferred to Google Earth image (Tele Atlas North America Inc., Menlo Park, CA, USA) using MapInfo Professional software version 7.8 (Pitney Bowes MapInfo Corporation, New York, NY, USA).

In mid-July 2007, 4 to 5 weeks following the well survey, 11 wells—10 previously identified as low-oxygen wells and one as normoxic—were selected for measurement of other gases in the well atmosphere at the deepest depth. The atmosphere was sampled using an air sampler pump with attached Tygon tubing that was lowered to the levels of 30 cm above the water surface. A 3 L airbag was connected to the pump for collecting the atmospheric sample. The sample was then analyzed using an ambient air analyzer (MIRAN 205B Series SapphIRe Portable Ambient Air Analyzer; Thermo Electron Corporation, Franklin, MA, USA), which has the ability to identify unknown airborne compounds using the ThermoMatch software option (Thermo Environmental Instruments, Inc., Franklin, MA, USA) with over 100 standard gases contained in its library, and subsequently also analyzed for its carbon dioxide content using an indoor air quality meter (IQ-410 Indoor Air Quality Meter; GrayWolf Sensing Solutions, Ltd, Annacotty, Ireland) fitted with a carbon dioxide sensor.

Data were computerized using EpiData version 3.1 (The EpiData Association, Odense M, Denmark) for data entry incorporating logic and range checks, and transferred to R statistical package version 2.7.1 (The R Foundation for Statistical Computing, Vienna, Austria) and Stata version 7 (StatCorp, College Station, TX, USA) for data exploration and cleaning, using cross tabulation and examination of summary statistics. The distribution of oxygen concentration with depth in the air column of each well was examined graphically. Wells were classified as low oxygen if oxygen measurement at any depth was <19.5%, and normoxic otherwise. Well characteristics, namely village of location, age, dimensions, construction, condition, surroundings, and type of water use, were compared between low-oxygen and normoxic wells using rank sum, chi-square, and Fisher's exact tests, as appropriate to the type and distribution of the data. Those variables showing some indication of differences (*p* < 0.25) were selected as candidate variables for initial inclusion in a multivariate logistic regression model, which was then refined by backward elimination guided by the change in log likelihood of successive models, to identify characteristics having an independent association with the low-oxygen status.

Approval for the study's confidentiality, ethics, and safety standards was granted by the Ethics Review Committee of the Faculty of Medicine, Prince of Songkla University, Songkhla, Thailand, in accordance with the institutional and national guidelines for protection of human beings.

## 3 Results

The general structure of agricultural wells in the study region is shown in Fig. 1. Deep artesian wells had generally been bored to a reported depth of up to 60 m, and a shallow well was dug around the artesian pipe to a depth of around 10 m. A pump was located at the bottom of the shallow well, and an engine was set up at ground level beside the well, which was used to drive the pump via a set of drive belts. Setting up the system, and cleaning and maintaining the pump all necessitated entering the well.

All wells were located in or at the edge of fields. Subsequent analysis was confined to the 253 wells that had an air column of at least 2 m at the time of observation, representing 85.8% of all wells visited. Each shallow well was constructed with a concrete casing using prefabricated pipe sections having internal diameters of 0.8–1.2 m and depth of 5.0–14.1 m (median 8.8 m) from the top of the surrounding wall. The lower end of the artesian pipe was reported to be at depths of between 12 m and 60 m (median 24 m) below ground level. Water was delivered to ground level from the pump via a 3-inch ID iron or polyvinyl chloride (PVC) pipe. The power source for the pump was most commonly (94%) a 9.5–15 (median 10.5) hp diesel-driven tillage tractor engine.

Wells were used for irrigating areas of 2–80 rai (median 23 rai; 1 rai = 0.16 ha), and the three most common months for irrigation were February, March, and April. The mean highest frequency of irrigation was 3.4 (SD 1.1) times per month for an average of 3.8 (1.7) days each time. Eighty-two wells (32.4%) had not been used for 1–2 years, and 44 wells (17.4%) had not been used for < 2 years. Twenty-eight wells (11.1%) had been entered in the previous year for maintenance and/or cleaning.

**Fig. 2:**
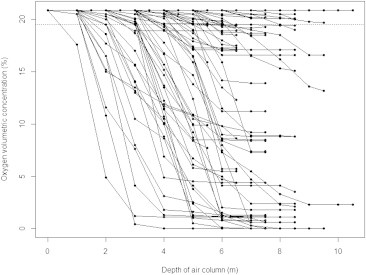
Oxygen volumetric concentration by depth in the air column of each measured well. Points indicating an oxygen concentration of 20.9% represent more than one well. The broken line marks an oxygen concentration of 19.5%.

Profiles of atmospheric oxygen volumetric concentration with depth in these 253 wells are shown in Fig. 2. In those wells in which oxygen measurement fell below 20.9%, oxygen content decreased with increasing depth, with the lowest content being recorded at the deepest depth. At the deepest depth, 67 wells (26.5%) had oxygen levels of <20.9% and 57 (22.5%) of <19.5% (“low-oxygen” wells). Of the wells, 11% in Phitsanulok province and 35% in Kamphaengphet province had a low-oxygen status. Among the five villages, the prevalence of low-oxygen wells ranged from 10% to 45% (Table 1).

Agitation of water in the 245 wells in which water was present caused a slight change in oxygen concentration (from −0.3% to +0.1%) in only 12 wells and a change of +1.3% in one well. No change was recorded in any of the other 232 wells containing water.

Age, dimensions, and characteristics of the wells according to oxygen status are shown in Tables 2 and 3, and their use according to oxygen status is shown in Table 4. Low-oxygen shallow wells were more commonly older, deeper to the bottom of the well and to the current water surface, with shallower current depth of water, of smaller diameter bore, fitted with a PVC water pipe, containing a rusted iron ladder, and covered. Low-oxygen wells were more frequently identified if there had been no rainfall in the previous 2 days. Low-oxygen wells were used for irrigating a larger area than normoxic wells, were more likely to have been left unused at least in the current year and, if not dry, were more likely to have water with a stale odor.

Well characteristics independently associated with a low-oxygen well status were identified using logistic regression modeling, in which low-oxygen status was the dependent variable. The following well characteristics were initially fitted to the model: village of location, age of the artesian well, age of the shallow well, internal diameter of the shallow well, depth of air column, water depth, type of delivery pipe, presence of a rusted iron ladder, presence of surrounding rubbish, presence of a cover over the well, area receiving irrigation, type and power of engine, months when the well was usually used for irrigation, number of years since the last use, time since the previous rainfall, frequency of entering the well in the previous year, and the type of soil surrounding the well.

After refinement of the model, five well characteristics retained a significant relationship with oxygen status (Table 5): depth to the water surface [odds ratio (OR) 6.25 (95% CI 0.76–51.3) and 13.8 (95% CI 1.65–110) for depths from ≥6 m to <8 m and from ≥8 m to ≤11 m, respectively; reference <6 m], water depth [OR 0.64 (95% CI 0.28–1.44] and 0.17 (95% CI 0.05–0.59) for depths from >1 m to <3 m and from ≥3 m to <8 m, respectively; reference ≤1 m], no rain in the previous 2 days [OR 7.44 (95% CI 2.13–26.0); reference rain within the previous 24 hours], having a cover [OR 3.95 (95% CI 1.22–12.8); reference not covered], and location in sandy soil [OR 3.72 (95% CI 1.56–8.87); reference other soil type]. After adjustment for these factors, geographic location of the well was not a significant predictor of low oxygen.

Hazardous gases, CO, H_2_S, and %LEL were detected in a small number of wells, but only at low levels (Table 6). Detectable CO and %LEL were significantly more common in low-oxygen wells. As these low levels were unlikely to be the explanation of the low oxygen content of the well atmosphere, samples of atmosphere from the bottom of 11 wells were collected to test for other unknown gases using the MIRAN SapphIRe Series Ambient Air Analyzer (Thermo Electron Corporation). However, in case of all samples, the equipment raised an automatic warning message that the result was interfered with by humidity and excessive levels of carbon dioxide, and the equipment, therefore, could not be used.

Instead, as carbon dioxide was suspected to be present in large proportions, an Indoor Air Quality Meter IQ-410 (GrayWolf Sensing Solutions) specific for carbon dioxide was employed to test the atmosphere at the deepest depth of these 11 wells at the same time as the oxygen content was measured using the EntryRAE model PGM-3000 gas monitor (RAE Systems). The results are shown in Table 7, in which wells are listed in an increasing order of oxygen content at the time of carbon dioxide measurement.

All 10 initially low-oxygen wells had increased oxygen levels at the time of these measurements, with three of them becoming normoxic, and the initially normoxic well remained so. Carbon dioxide levels were above the range of ±50 ppm accuracy, quoted in the equipment specifications as being up to 10,000 ppm, in seven of the 10 initially low-oxygen wells that remained low-oxygen: >80,000 ppm in five, and 26,000 ppm and 65,000 ppm in the other two. Carbon dioxide levels of the three wells that were now found to be normoxic had carbon dioxide levels of 460 ppm, 490 ppm, and 2,230 ppm, while the originally normoxic well that remained so had a carbon dioxide level of 345 ppm, only slightly higher than the carbon dioxide level of 254 ppm measured in the ambient air at ground level.

## 4 Discussion

In this survey of agricultural shallow wells in the lower north of Thailand, one in five wells in the study area had oxygen levels that would be harmful to a person entering the well, whereas the other detected gases, carbon monoxide, hydrogen sulfide, and explosive gases, present in some wells, were not at high enough levels to pose an immediate hazard. Oxygen concentrations <16% are considered to be extremely hazardous, and this condition was found in 39 (15.4%) of the total of 253 wells or in 68.4% of the 57 low-oxygen wells. Exposure to this low level of oxygen leads to increased heart rate; disturbed respiration; impaired thinking, attention, and judgment; and reduced coordination. At an oxygen level of <10% unconsciousness may occur and <6% death occurs within minutes [11]. The prevalence of low-oxygen wells in this survey was somewhat higher than that of the previous survey in the same general area (16%), conducted by the Bureau of Occupational and Environmental Diseases, but it was evident from the current survey that the prevalence also depended on the particular area surveyed [10,12].

A reduction of oxygen content with increasing depth in the air column of low-oxygen wells is consistent with the utilization or displacement of oxygen within the well, coupled with a restricted bulk airflow vertically in the air column and/or a longer diffusion pathway for equilibration with the ambient air above [13,14].

Thus, in the multivariate model, depth of a well to the water level was found to be an independent predictor of whether the well was a low-oxygen one. The independent association of a well having a cover with the low-oxygen status can also be explained by the cover restricting bulk airflow in the air column. Rainfall (in noncovered wells at least) may be expected to encourage air currents within the air column, thereby adding to bulk airflow as well as to diffusion within the well atmosphere, and this might be the explanation for the negative association between low-oxygen status and recent rainfall.

Independent of the depth of the air column to the water surface, the depth of water itself was negatively associated with the probability of the well having a low-oxygen condition. A plausible explanation for this relationship is not evident, but in some way the water may be acting as a buffer against large deviations in the composition of the well atmosphere from that of ambient air.

The reason why location of the well in a site of sandy soil should be associated with the low-oxygen status is at present not understood; indeed, it is not known whether the relationship is based on very local effects, or whether soil composition reflects deeper geological characteristics that can affect oxygen utilization or production of other gases leading to oxygen displacement. However, 87% of the wells sited in sandy soil were located in Kamphaengphet province, where almost all wells had been left unused for at least 1 year, so that, despite the lack of statistical significance of well use in the model, the association of the low-oxygen status with sandy soil may have masked an underlying association between not using a well and the development of the low-oxygen status.

Carbon dioxide levels in the 7 low-oxygen wells in July ranged from 26,000 ppm (2.6%) to over 80,000 ppm (>8.0%) (assuming that the equipment reading still had some validity at these levels). Such gas content strongly suggests that the oxygen in the atmosphere was being replaced by carbon dioxide, although the possibility that carbon dioxide was simply being added to the atmosphere cannot be ruled out. However, for the low oxygen levels to be the result of displacement of air, extremely large volumes of carbon dioxide would have been required. For instance, to decrease oxygen volumetric concentration to 10% would require a carbon dioxide content of about 52% or 520,000 ppm. By contrast, to lower oxygen concentration from 20.9% to 10% through replacement with carbon dioxide would theoretically require a carbon dioxide concentration of only 109,000 ppm (10.9%) [15]. Indeed, the 65,000 ppm carbon dioxide recorded in the well with 13.0% oxygen and the 26,000 ppm carbon dioxide in the well with 17.8% oxygen correspond closely to the theoretical carbon dioxide contents of 79,000 ppm and 31,000 ppm, respectively, if it is considered that oxygen was replaced by carbon dioxide; carbon dioxide was possibly produced by the vegetation decay processes taking place inside the well. Furthermore, the higher density of carbon dioxide than that of air at ambient temperatures means that, having entered a well, carbon dioxide is likely to remain at the bottom of the air column. Such a pattern is consistent with the steep depth gradient of oxygen concentration found in the low-oxygen wells in this setting.

The level of carbon dioxide that is immediately dangerous to life or health is reported to be 40,000 ppm [16]. If the mechanism of oxygen depletion in these shallow wells does indeed involve its replacement by carbon dioxide, then the carbon dioxide levels are likely to exceed the immediately dangerous to life or health level in wells having an oxygen concentration of <16.9%, and thereby pose an additional hazard superimposed on that resulting from the low oxygen content. Such may have been the cause of the rapid fatalities that occurred in Kamphaengphet province in 2006. Similar conditions have been reported in other confined spaced fatalities [2,17-22].

Interpretation of the relationship between the levels of oxygen and those of carbon dioxide and/or other gases in the well atmosphere is limited by the small number of gases tested for and the small subset of wells used for analysis of carbon dioxide. In addition, oxygen levels in this subset differed somewhat from the levels measured in the Survey conducted 4 to 5 weeks earlier, indicating that oxygen levels may change over time, leading to some reservations over the predictors identified in the cross-sectional survey. A time-series study monitoring a range of gases in well atmospheres may help provide more valid information on predictors of hazardous atmospheric composition.

In conclusion, oxygen concentrations in the atmosphere of the agricultural shallow wells in the study area varied widely from well to well, decreasing with depth within many wells. Low-oxygen wells comprised over one-fifth of all wells investigated, and the low-oxygen condition was more likely among wells having a deeper air column, a shallower water depth, and a cover. Some evidence points to the likelihood that elevated carbon dioxide levels may replace oxygen in low-oxygen wells. The prevalence of wells having hazardous atmospheres and the level of hazard posed indicate a health hazard of sufficient magnitude to warrant intervention to reduce the risks to local farmers who continue to use well water for irrigating paddy fields.

## Conflicts of interest

The authors declare that they have no conflicts of interest.

## Figures and Tables

**Table 1:** Number of wells in the study sites, according to well atmosphere oxygen status

Site (No. of observed wells)	No. of measured wells (row %)∗				*p*†
	Total	Normoxic	Low O_2_		
		≥16.0%	<19.5–≥16.0%	<16.0%	
Kamphaengphet					<0.001
Village 1 (41)	32	26 (81.2)	2 (6.3)	4 (12.5)	
Village 2 (69)	53	29 (54.7)	4 (7.5)	20 (37.7)	
Village 12 (48)	35	23 (65.7)	1 (2.9)	11 (31.4)	
Phitsanulok					
Village 6 (51)	49	42 (85.7)	4 (8.2)	3 (6.1)	
Village 7 (86)	84	76 (90.5)	7 (8.3)	1 (1.2)	
Total (295)	253 (100)	196 (77.5)	18 (7.1)	39 (15.4)	

∗ Forty-two wells had insufficient air column for measurement.

† Chi-square test comparing normoxic and low-oxygen wells of all villages.

**Table 2:** Age and dimension characteristics (median and interquartile range) of wells, according to well atmosphere oxygen status

Characteristic	Normoxic wells (*n* = 196)	Low-oxygen wells (*n* = 57)	*p*∗
Artesian well age (y)	16 (10, 20)	15 (10, 17)	0.011
Shallow well age (y)	15 (10, 20)	10 (9, 16)	0.003
Artesian well depth (m)	24 (20, 28)	24 (19, 36)	0.692
Shallow well depth† (m)	8.5 (8, 10)	9.5 (8, 10.5)	0.035
Depth to water surface† (m)	6.6 (5, 8)	8 (7, 8.5)	<0.001
Current water depth (m)	2.3 (1, 3.8)	1 (0.9, 2)	<0.001
Diameter (m)	1.1 (0.9, 1.1)	0.9 (0.9, 1)	<0.001
Height of surrounding wall (m)	0.3 (0.2, 0.4)	0.2 (0.1, 0.3)	0.166

∗ Rank sum test.

† Measured from the top edge of the surrounding wall.

**Table 3:** Construction, condition, surroundings, and use characteristics of wells, according to well atmosphere oxygen status

Characteristic	No. of wells (column %)		*p*
	Normoxic (*n* = 196)	Low O_2_ (*n* = 57)	
Pipe type			
No pipe	14 (7.1)	4 (7.0)	0.018∗
Iron	155 (79.1)	36 (63.2)	
PVC	27 (13.8)	17 (29.8)	
Iron ladder	58 (29.6)	21 (36.8)	0.380∗
Cement casing	172 (87.8)	48 (84.2)	0.634∗
Leakage of water	89 (45.4)	23 (40.4)	0.600∗
Rust occurrence			
On ladder	31 (15.8)	17 (29.8)	0.029∗
On pipe	73 (37.2)	17 (29.8)	0.383∗
On leaked water	16 (8.2)	2 (3.5)	0.379†
Surroundings			
Large tree (>2 m)	131 (66.8)	36 (63.2)	0.721∗
Small tree (<2 m)	75 (38.3)	24 (42.1)	0.712∗
Hut	61 (31.1)	19 (33.3)	0.877∗
Rubbish, oil	18 (9.2)	4 (7.0)	0.791†
Roof	8 (4.1)	1 (1.7)	0.688†
Cover (>80%)	9 (4.6)	10 (17.5)	0.003†
Soil type			
Loam	168 (85.7)	36 (63.2)	<0.001†
Sandy loam	16 (8.2)	15 (26.3)	
Sandy	3 (1.5)	5 (8.8)	
Sandy clay	6 (3.1)	0 (0)	
Clay	1 (0.5)	1 (1.8)	
Clay loam	2 (1)	0 (0)	
Period since last rain (d)			
0	57 (29.1)	11 (19.3)	<0.001∗
1	131 (66.8)	31 (54.4)	
2	8 (4.1)	15 (26.3)	
Water‡			
Color—not clear	25 (13.2)	7 (12.5)	0.887∗
Odor—not fresh	39 (20.6)	19 (33.9)	0.040∗

PVC, polyvinyl chloride.

∗ Chi-square test.

† Fisher exact test.

‡ A total of 189 normoxic and 56 low-oxygen wells contained water.

**Table 4:** Well use according to well atmosphere oxygen status

Characteristic	No. of wells (column %)		*p*
	Normoxic (*n* = 196)	Low O_2_ (*n* = 57)	
Area of irrigation (rai)	20 (15, 30)	28 (20, 40)	0.021∗
Frequency of irrigation (time per month)	3 (3, 4)	3 (3, 4)	0.140∗
Duration of irrigation each time (d)	3 (3, 4)	3 (3, 5)	0.289∗
Cessation of use (y)			
Use in current year	112 (57.1)	15 (26.3)	<0.001†
Stopped using ≤2 y	55 (28.1)	27 (47.4)	
Stopped using >2 y	29 (14.8)	15 (26.3)	
Well ever having been entered			
No	170 (86.7)	55 (96.5)	0.068†
Yes	26 (13.3)	2 (3.5)	
Used for irrigation in the previous week			
No	190 (96.9)	53 (93.0)	0.239‡
Yes	6 (3.1)	4 (7.0)	

Data are presented as *n* (%) or median, IQR. IQR, interquartile range.

∗ Rank sum test.

† Chi-square test.

‡ Fisher's exact test.

**Table 5:** Multivariate logistic model of low-oxygen atmosphere at the deepest depth

Factor	Level	Odds ratio	95% CI	*p*∗
Depth to water surface (m)	<6	1		0.003
	≥6–<8	6.25	0.76–51.3	
	≥8–≤11	13.5	1.65–110.1	
Depth of water (m)	≤1	1		0.008
	>1–<3	0.64	0.28–1.44	
	≥3–<8	0.17	0.05–0.59	
Time since last rain (d)	<1	1		0.001
	>1–≤2	0.95	0.40–2.26	
	>2	7.44	2.13–26.0	
Well covered	No	1		0.021
	Yes	3.95	1.22–12.8	
Surrounding soil with sandy component	No	1		0.003
	Yes	3.72	1.56–8.87	

CI, confidence interval.

∗ Likelihood ratio test.

**Table 6:** Other detected gases in well atmosphere, according to well atmosphere oxygen status

Gas (range among wells in which the gas was detected)	No. of wells (column %)		*p*
	Normoxic (*n* = 196)	Low O_2_ (*n* = 57)	
Carbon monoxide (0.4–5.7 ppm)	39 (19.9)	36 (63.2)	<0.001∗
Hydrogen sulfide (0.5–0.6 ppm)	3 (1.5)	3 (5.3)	0.130†
Lower explosive limit (0.3–3%)	0 (0.0)	7 (12.3)	<0.001†

∗ Chi-square test.

† Fisher's exact test.

**Table 7:** Carbon dioxide and oxygen levels in the subset of 11 wells

Well ID	Location∗	Air column depth (m)	Oxygen (%) at deepest depth		CO_2_ (ppm)	
			First survey June 10–16	Current July 19–20	Surrounding air at ground level	At deepest depth of air column in well
42	K	9.0	0.6	2.4	Nm	>80,000
50	K	11.0	2.3	6.1	275	>80,000
41	K	9.3	0.6	6.2	Nm	>80,000
52	K	10.0	0.0	7.6	290	>80,000
18	K	9.0	2.1	9.5	Nm	>80,000
124	P	9.0	3.5	13.0	287	65,000
15	K	8.0	7.4	17.8	Nm	26,000
49	K	6.8	5.5	20.9	222	2,230
24	K	7.0	17.1	20.9	288	490
21	K	8.2	0.6	20.9	228	460
22	K	8.0	20.9	20.9	254	345

K, Kamphaengphet; Nm, not measured; P, Phitsanulok.

∗ Location in K or P province.
